# Metabolic Promiscuity of an Orphan Small Alarmone Hydrolase Facilitates Bacterial Environmental Adaptation

**DOI:** 10.1128/mbio.02422-22

**Published:** 2022-12-06

**Authors:** Danny K. Fung, Kaihong Bai, Jin Yang, Xiaoli Xu, David M. Stevenson, Daniel Amador-Noguez, Laixin Luo, Jue D. Wang

**Affiliations:** a Department of Bacteriology, University of Wisconsin—Madison, Madison, Wisconsin, USA; b Department of Plant Pathology, China Agricultural University, Beijing Key Laboratory of Seed Disease Testing and Control, Beijing, People’s Republic of China; Max-Planck-Institut fur terrestrische Mikrobiologie

**Keywords:** (p)ppGpp, NADH, nucleotide metabolism, plant pathogens, stress response

## Abstract

Small alarmone hydrolases (SAHs) are alarmone metabolizing enzymes found in both metazoans and bacteria. In metazoans, the SAH homolog Mesh1 is reported to function in cofactor metabolism by hydrolyzing NADPH to NADH. In bacteria, SAHs are often identified in genomes with toxic alarmone synthetases for self-resistance. Here, we characterized a bacterial orphan SAH, i.e., without a toxic alarmone synthetase, in the phytopathogen Xanthomonas campestris pv. *campestris* (*Xcc*SAH) and found that it metabolizes both cellular alarmones and cofactors. *In vitro*, *Xcc*SAH displays abilities to hydrolyze multiple nucleotides, including pppGpp, ppGpp, pGpp, pppApp, and NADPH. *In vivo*, X. campestris pv. *campestris* cells lacking *sah* accumulated higher levels of cellular (pp)pGpp and NADPH compared to wild-type cells upon amino acid starvation. In addition, X. campestris pv. *campestris* mutants lacking *sah* were more sensitive to killing by Pseudomonas during interbacterial competition. Interestingly, loss of *sah* also resulted in reduced growth in amino acid-replete medium, a condition that did not induce (pp)pGpp or pppApp accumulation. Further metabolomic characterization revealed strong depletion of NADH levels in the X. campestris pv. *campestris* mutant lacking *sah*, suggesting that NADPH/NADH regulation is an evolutionarily conserved function of both bacterial and metazoan SAHs and Mesh1. Overall, our work demonstrates a regulatory role of bacterial SAHs as tuners of stress responses and metabolism, beyond functioning as antitoxins.

## INTRODUCTION

To cope with changing environments, most bacteria produce nucleotide signaling molecules, such as the alarmone guanosine tetra/pentaphosphate [(p)ppGpp] (1) to survive various stresses (2, 3). (p)ppGpp concentrations can range from micromolar to low millimolar levels in cells (4, 5) to directly regulate many central cellular processes, including transcription, translation, DNA replication, and purine metabolism (6–11). Apart from (p)ppGpp, nucleotides such as pGpp (12) and AppppA (13) can also function as alarmones, while (p)ppApp has been recently reported as a toxin (14).

Cellular (p)ppGpp concentration is dictated by its turnover via dedicated alarmone synthetases and hydrolases (15, 16), highlighting their fundamental importance in alarmone signaling. Alarmone synthetases and hydrolases belong to the RelA/SpoT homologs (RSHs) superfamily (16), which is further classified into multidomain “long” RSHs or single-domain homologs known as small alarmone synthetases (SASs), or small alarmone hydrolases (SAHs) (10, 15). Long RSHs include the synthetase RelA (1) and the bifunctional synthetase and hydrolase Rel (17) and SpoT (18), which produce (p)ppGpp and in some cases pGpp (19). Examples of SASs include SasA (RelP), SasB (RelQ), and RelV (20–23), which produce (p)ppGpp and in some cases pGpp (24) or (p)ppApp (25). In addition, SAS homologs have been reported to be growth-inhibiting toxins in contact-dependent inhibition or phage defense, by producing (p)ppApp to a toxic high concentration (14, 26) or by pyrophosphorylating tRNA to inactivate its essential function in protein translation (27).

In contrast to long RSHs or SASs, SAHs contain only the alarmone hydrolase domain. SAHs are classified into seven different subgroups (16): paSpo, pbcSpo, pbcSpo2, Mesh1, Mesh1-L, rickSpo, and divSpo. SAHs were first identified in metazoans as Mesh1 (28) and more recently in bacteria (26, 29–31). Mesh1 has been reported to hydrolyze ppGpp (28, 30) and the metabolic cofactor NADPH (32, 33). Mesh1 can regulate development, nutritional stress response, ferroptosis, sleep behavior, endoplasmic reticulum proteostasis, and cell viability in Drosophila melanogaster, Caenorhabditis elegans, and human cells (33–37). In contrast, much less is understood about SAHs in bacteria. Recent reports showed that SAHs can function as self-resistance antitoxins against SAS toxins such as Tas1 (14) and FaRel and FaRel2 (26) by hydrolyzing alarmones such as (p)ppApp (29) or by depyrophosphorylation of pyrophosphorylated tRNA (27). However, the physiological roles of bacterial SAHs beyond antagonizing toxins remain uncharacterized.

**FIG 1:**
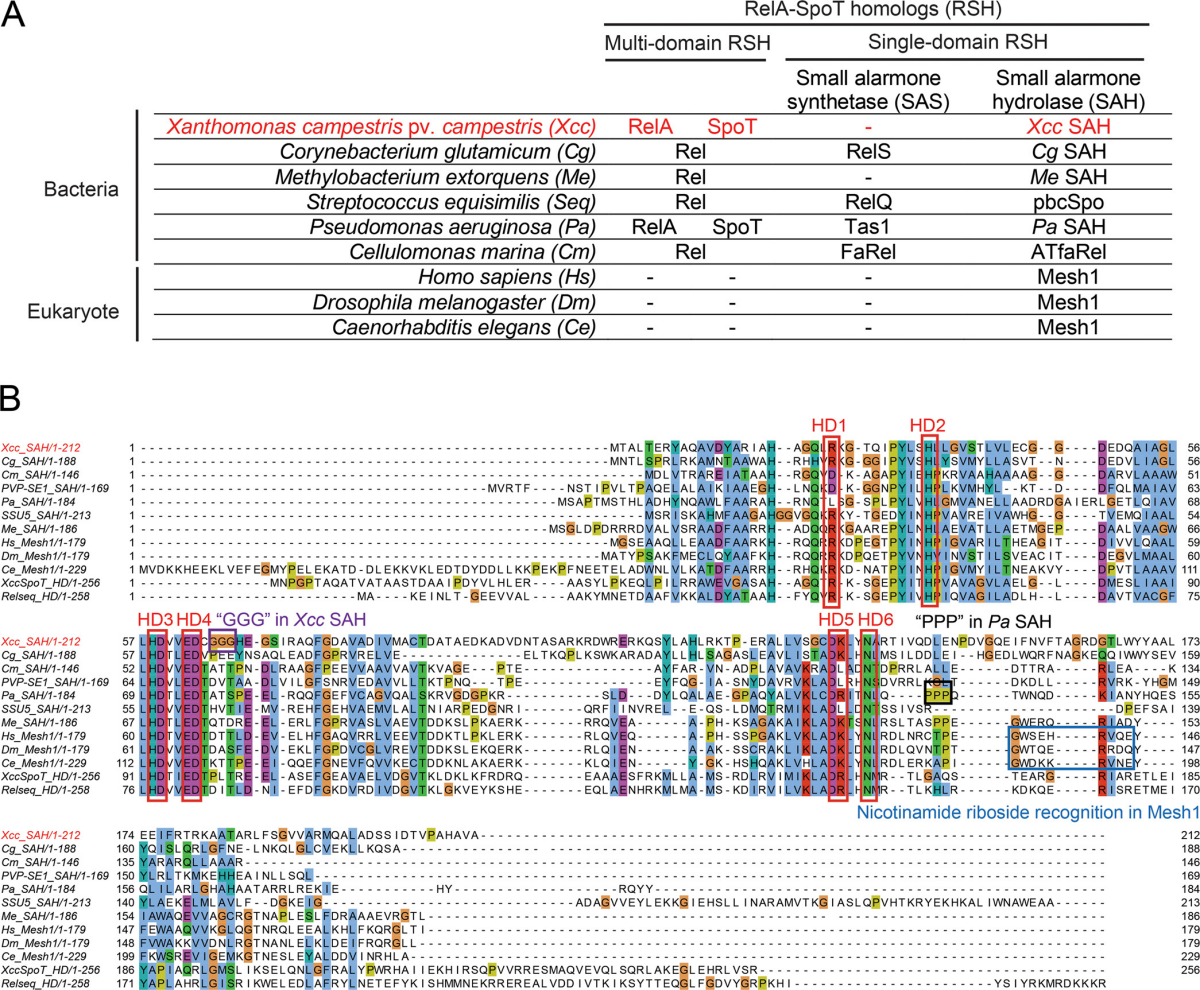
Phylogeny and amino acid sequence comparison of *Xcc*SAH to various (p)ppGpp hydrolase homologs. (A) Alarmone metabolizing enzymes in X. campestris pv. *campestris* and other bacterial and eukaryotic species. RelA-SpoT homologs (RSH) comprise a large family of multidomain or single-domain alarmone synthetases and hydrolases. In addition to multidomain RSH enzymes RelA and SpoT for (p)ppGpp synthesis and hydrolysis, *Xanthomonas* species such as Xanthomonas campestris pv. *campestris* contain a putative orphan small alarmone hydrolase (SAH). Hyd-Syn, bifunctional hydrolase and synthetase. (B) Amino acid sequence alignment of *Xcc*SAH and other RSH hydrolases. Red boxes indicate conserved alarmone hydrolase domain (HD) motifs (HD1 to -6), according to a previous report (42). Blue box indicates nicotinamide riboside recognition sites reported in Mesh1 (32). Black box indicates the characteristic triple-proline (P138 to -140) region in P. aeruginosa SAH (29). Purple box indicates a unique triple-glycine (G65 to -67) region in *Xcc*SAH.

Xanthomonas campestris is a pathogenic plant bacterium with a broad host range (38–40). *Xanthomonas* carries two multidomain RSH enzymes that are responsible for (p)ppGpp production and hydrolysis (Fig. 1A, Table 1). Intriguingly, the majority of *Xanthomonas* species also carry a putative SAH (Fig. 1A, Table 1) belonging to the Mesh1-L subgroup (16), although no toxic alarmone synthetases coexist in the genome. Therefore, we characterized this orphan SAH from the model pathogen X. campestris pv. *campestris* *in vitro* under laboratory conditions and in its native environments. We found that the *Xcc*SAH was highly promiscuous and hydrolyzed multiple alarmones and the cofactor NADPH. In contrast to the multidomain RSHs, which are required for X. campestris pv. *campestris* pathogenesis, as we reported previously (41), *Xcc*SAH is not required for leaf infection but contributes to various aspects of X. campestris pv. *campestris* physiology, including modulation of (pp)pGpp levels during starvation, survival against competing Pseudomonas species, as well as metabolism of cellular NADH and growth under defined conditions. Our results provide an example of how SAHs can play multiple biological roles in bacteria beyond functioning as antitoxins, suggesting a unifying theme of SAHs as multifunctional physiology regulators in both bacteria and metazoans.

**TABLE 1:** RSH homologs in *Xanthomonas* species deduced from sequenced genomes^*a*^

GCF_numbers	TaxID	Organism	RSHs
GCF_000087965.2	380358	Xanthomonas albilineans GPE PC73	SpoT[HS], RelA[HS]
GCF_000348585.1	1304892	Xanthomonas axonopodis Xac29-1	SpoT[HS], Mesh1-L
GCF_000364685.1	1240726	*Xanthomonas* sp. SHU166	Mesh1-L, SpoT[HS]
GCF_000963005.1	1583099	*Xanthomonas* sp. GPE 39	SpoT[HS], RelA[HS]
GCF_000963215.1	1588031	*Xanthomonas* sp. MUS 060	SpoT[HS], RelA[HS]
GCF_001306995.1	1732019	*Xanthomonas* sp. Mitacek01	SpoT[HS], RelA[S]
GCF_001423495.1	1736270	*Xanthomonas* sp. Leaf131	SpoT[HS], RelA[S]
GCF_002023005.1	64187	Xanthomonas oryzae pv. *oryzae*	RelA[HS], SpoT[HS]
GCF_900018785.1	1720302	*Xanthomonas massiliensis*	SpoT[HS], RelA[S]
GCF_000159795.2	559736	Xanthomonas vasicola pv. *vasculorum* NCPPB 702	SpoT[HS], RelA[S], Mesh1-L
GCF_000192045.2	925776	Xanthomonas perforans 91-118	RelA[S], Mesh1-L, SpoT[HS]
GCF_000225915.1	981368	Xanthomonas axonopodis pv. *citrumelo* F1	Mesh1-L, RelA[S], SpoT[HS]
GCF_000364645.1	1248412	*Xanthomonas* sp. SHU308	Mesh1-L, RelA[S], SpoT[HS]
GCF_000364665.1	1240783	*Xanthomonas* sp. SHU199	Mesh1-L, RelA[S], SpoT[HS]
GCF_000401255.1	1321368	Xanthomonas maliensis	Mesh1-L, SpoT[HS], RelA[S]
GCF_000764855.1	1885902	*Xanthomonas cannabis* pv*. phaseoli*	SpoT[HS], RelA[HS], Mesh1-L
GCF_001010415.1	1440766	Xanthomonas pisi DSM 18956	SpoT[HS], Mesh1-L, RelA[HS]
GCF_001186465.1	340	Xanthomonas campestris pv. *campestris*	SpoT[HS], Mesh1-L, RelA[HS]
GCF_001237985.1	195709	Xanthomonas arboricola pv. *juglandis*	RelA[S], SpoT[HS], Mesh1-L
GCF_001423585.1	1736275	*Xanthomonas* sp. Leaf148	RelA[HS], SpoT[HS], Mesh1-L
GCF_001610795.1	76802	Xanthomonas fuscans subsp. *aurantifolii*	RelA[S], SpoT[HS], Mesh1-L
GCF_001642575.1	1843580	Xanthomonas floridensis	Mesh1-L, RelA[S], SpoT[HS]
GCF_001660815.1	1843581	Xanthomonas nasturtii	Mesh1-L, RelA[S], SpoT[HS]
GCF_001908725.1	925775	Xanthomonas vesicatoria ATCC 35937	RelA[HS], Mesh1-L, SpoT[HS]
GCF_001908775.1	90270	Xanthomonas gardneri	Mesh1-L, RelA[HS], SpoT[HS]
GCF_001908795.1	456327	Xanthomonas euvesicatoria	Mesh1-L, RelA[S], SpoT[HS]
GCF_002285515.1	56454	Xanthomonas hortorum	Mesh1-L, RelA[HS], SpoT[HS]
GCF_002759355.1	473423	Xanthomonas citri pv*. phaseoli* var*. fuscans*	SpoT[HS], RelA[S], Mesh1-L
GCF_900092025.1	56449	Xanthomonas bromi	SpoT[HS], Mesh1-L, RelA[HS]
GCF_900094325.1	1261556	Xanthomonas translucens pv*. translucens DSM 18974*	RelA[S], SpoT[HS], Mesh1-L
GCF_900143175.1	305959	*Xanthomonas retroflexus*	SpoT[HS], RelA[S], PbcSpo[H]
GCF_900183985.1	48664	Xanthomonas fragariae	Mesh1-L, RelA[S], SpoT[HS]
GCF_000454545.1	1219375	Xanthomonas cassavae CFBP 464*2*	SpoT[HS], RelA[S], CaPRel[S], Mesh1-L
GCF_000815185.1	56458	Xanthomonas sacchari	MixRel[S], Mesh1-L, SpoT[HS], RelA[S]
GCF_001043115.1	1775876	*Xanthomonas* sp. NCPPB1128	CaPRel[S], SpoT[HS], Mesh1-L, RelA[S]
GCF_002759155.1	317013	Xanthomonas phaseoli pv. *phaseoli*	PbcSpo[H], Mesh1-L, RelA[HS], SpoT[HS]

a Original data from reference 26. Species shaded in blue contain a putative alarmone hydrolase relabeled as Mesh1-L (16) (originally labeled MixSpo[H] in reference 26).

## RESULTS

### Identification of an orphan SAH in *Xanthomonas*.

In the *Xanthomonas* model organism X. campestris pv. *campestris* 8004, we identified a single-domain open reading frame (XC_RS15930, designated *Xcc*SAH hereafter) homologous to the hydrolase domain of the bifunctional synthetase hydrolase SpoT (Table 2). Compared to Mesh1 in metazoans (28, 32), *Xcc*SAH belongs to the phylogenetically distinct sister group known as Mesh1-L (16). Unlike Mesh1, *Xcc*SAH does not have a well-defined nicotinamide ribose interaction motif, which has been proposed to allow NADPH hydrolysis (32) (Fig. 1B). In general, *Xcc*SAH contains key domain architectures of an alarmone hydrolase, with conserved residues that are critical for nucleotide base coordination, metal coordination, and nucleophilic attack of the 3′-phosphates (Fig. 1B, HD1-6) (42). These features suggest that *Xcc*SAH is likely a functional alarmone hydrolase.

**TABLE 2:** Proteins exhibiting homology with the (p)ppGpp hydrolase domains of RelA and SpoT in X. campestris pv. *campestris* 8004

Query sequence	Gene ID (NCBI)	Sequence identity (%)	Function annotation	*P* value
RelA hydrolase domain	XC_RS05900 (*relA*)	100	Bifunctional (p)ppGpp	1.00E−97
	XC_RS04795 (*spoT*)	36.43	Synthetase/guanosine-3′,5′-bis(diphosphate)	3.00E−10
	XC_RS10860	35.85	β-*N*-acetylglucosaminidase	0.53
	XC_RS21410	32.26	ATP-dependent DNA helicase Rep	0.92
	XC_RS04970	44.12	Assimilatory sulfite reductase (NADPH)	4.9
	XC_RS00080	33.87	Hypothetical protein	6.8
	XC_RS00850	28.36	Aminotransferase	7.3
SpoT hydrolase domain	XC_RS04795 (*spoT*)	100	Synthetase/guanosine-3′,5′-bis(diphosphate)	6.00E−98
	XC_RS15930 (*sah*)	30.53	HD domain-containing protein	1.00E−12
	XC_RS05900 (*relA*)	36.43	Bifunctional (p)ppGpp	9.00E−11
	XC_RS06855	26.15	OsmC family peroxiredoxin	2.5
	XC_RS09145	32.43	GGDEF-domain containing protein	2.6
	XC_RS13715	38.71	Methionine synthase	6.9
	XC_RS18395	23.64	Cystathionine beta-synthase	8

### *Xcc*SAH hydrolyzes multiple alarmones and NADPH with different efficiencies.

**FIG 2:**
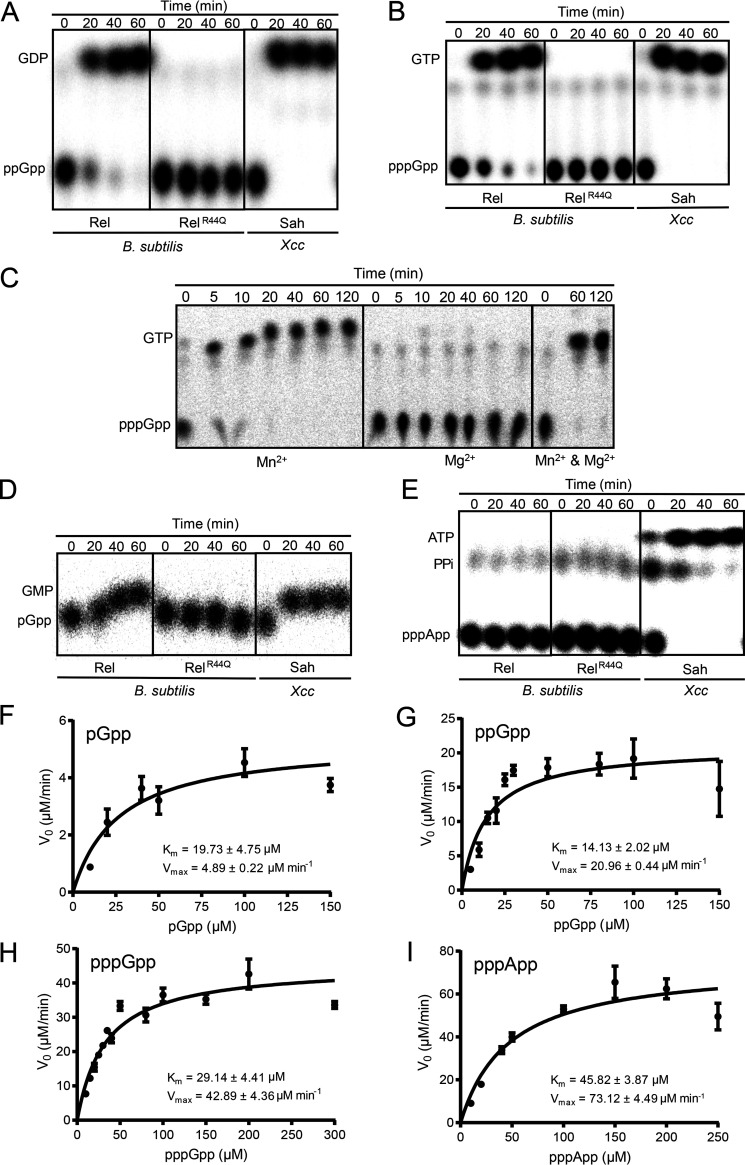
*Xcc*SAH hydrolyzes (p)ppGpp, pGpp, and pppApp *in vitro*. (A and B) TLC analysis of (p)ppGpp hydrolysis by B. subtilis Rel (B. subtilis bifunctional synthetase-hydrolase Rel), B. subtilis Rel^R44Q^ (hydrolase-defective variant of B. subtilis Rel), or *Xcc*SAH. Hydrolysis reactions were carried out at 37°C in 20 mM Tris-HCl (pH 7.5), 1 mM MnCl_2_, ^32^P-radiolabeled nucleotides {[5′-α-^32^P](pp)pGpp or [5′-γ-^32^P, 3′-β-^32^P]pppApp}, and 240 nM purified enzymes. Samples were quenched with 2 M formic acid at different times, as indicated. (C) TLC analysis of pppGpp hydrolysis by *Xcc*SAH using either 1 mM MnCl_2_ or 10 mM MgCl_2_ or both metals. (D and E) TLC analysis of pGpp (D) or pppApp (E) hydrolysis by *Xcc*SAH. PPi (inorganic pyrophosphate) is released from the cleavage of 3′-β-^32^P label from [5′-γ-^32^P, 3′-β-^32^P]pppApp. (F to I) Hydrolysis kinetics of pGpp (F), ppGpp (G), pppGpp (H), and pppApp (I) by *Xcc*SAH. Hydrolysis reactions were carried out at 37°C in 20 mM Tris-HCl (pH 7.5), 1 mM MnCl_2_, ^32^P-radiolabeled nucleotides, and 25 nM (for pppApp) or 50 nM (for other nucleotides) *Xcc*SAH. Samples were quenched with 2 M formic acid at different times as indicated, followed by TLC analysis and quantitation. Data were fitted by nonlinear regression to the Michaelis-Menten equation. Error bars indicate standard deviations from three independent experiments.

To examine the biochemical properties of *Xcc*SAH, we recombinantly expressed and purified *Xcc*SAH from Escherichia coli, performed *in vitro* enzymatic assays by incubating *Xcc*SAH with radiolabeled alarmones, and monitored the products of the reaction using thin-layer chromatography (TLC) (Fig. 2A to E). For comparison, the bifunctional RSH enzyme Rel (historically called RelA) (17, 43) from the soil bacterium Bacillus subtilis, which has (p)ppGpp hydrolysis activity, and its hydrolase-defective variant Rel^R44Q^ were used as controls. Similar to B. subtilis Rel, *Xcc*SAH efficiently hydrolyzed ppGpp and pppGpp to GDP and GTP, respectively (Fig. 2A and B) and required manganese (Mn^2+^) for its activity (Fig. 2C). This metal selectivity was consistent with other previously reported (p)ppGpp hydrolases (29, 44). We found that B. subtilis Rel could also hydrolyze pGpp to GMP (Fig. 2D) but had no detectable pppApp hydrolysis activity (Fig. 2E). In contrast, *Xcc*SAH hydrolyzed all alarmones tested, including pGpp and pppApp, to GMP and ATP, respectively (Fig. 2D and E). For pppApp hydrolysis, the additional spot under ATP is ^32^P-labeled pyrophosphate, since the pppApp was labeled with both 5′-γ-^32^P and 3′-β-^32^P (Fig. 2E). Overall, our results indicated that *Xcc*SAH has an expanded substrate spectrum compared to long RSH enzymes.

We next studied the enzyme kinetics of *Xcc*SAH on the hydrolysis of pGpp, ppGpp, pppGpp, and pppApp using Michaelis-Menten kinetics (Fig. 2F to I and Table 3). Among the four substrates, *Xcc*SAH hydrolyzed pppApp most efficiently, with a *k*_cat_ of 48.75 ± 2.99 s^−1^ (mean ± standard deviation) and *k*_cat_/*K_m_* ratio (catalytic efficiency) of (10.67 ± 0.75) × 10^5^ s^−1^ M^−1^ (Table 3). pppGpp and ppGpp hydrolysis was ~50% less efficient than pppApp, with *k*_cat_/*K_m_* ratios of (5.00 ± 0.82) × 10^5^ s^−1^ M^−1^ for ppGpp and (4.93 ± 0.25) × 10^5^ s^−1^ M^−1^ for pppGpp (Table 3). Although *Xcc*SAH had higher affinities to pppGpp, ppGpp, and pGpp than pppApp (*K_m_* values of 29.14 ± 4.41 μM, 14.13 ± 2.02 μM, 19.73 ± 4.75 μM, and 45.82 ± 3.87 μM, respectively), the rate of hydrolysis was faster for pppApp, followed by pppGpp, ppGpp, and pGpp in decreasing order (*k*_cat_ of 48.75 ± 2.99 s^−1^,14.30 ± 1.45 s^−1^, 6.99 ± 0.15 s^−1^, and 1.63 ± 0.07 s^−1^, respectively) (Fig. 2F to I, Table 3). These findings suggest that the nucleotide hydrolysis efficiency of *Xcc*SAH is positively associated with the number of 5′-phosphates of the nucleotide [e.g., (p)ppGpp > pGpp] and to some extent the identity of the nucleobase (e.g., A > G).

**TABLE 3:** Kinetics of alarmone and cofactor hydrolysis by *Xcc*SAH^*a*^

Protein	Substrate	*V*_max_ (μM/min)	*K_m_* (μM)	*k*_cat_ (s^−1^)	*k*_cat_/*K_m_* (s^−1^ M^−1^)
Alarmone	pGpp	4.89 ± 0.22	19.73 ± 4.75	1.63 ± 0.07	(0.86 ± 0.18) × 10^5^
	ppGpp	20.96 ± 0.44	14.13 ± 2.02	6.99 ± 0.15	(5.00 ± 0.82) × 10^5^
	pppGpp	42.89 ± 4.36	29.14 ± 4.41	14.30 ± 1.45	(4.93 ± 0.25) × 10^5^
	pppApp	73.12 ± 4.49	45.82 ± 3.87	48.75 ± 2.99	(10.67 ± 0.75) × 10^5^
Cofactor	NADPH	2.88 ± 0.26	132.2 ± 39.09	0.048 ± 0.004	(3.63 ± 0.20) × 10^2^

a Calculations for SAH are based on data presented in Fig. 2 and 3. *V*_max_ and *K_m_* were calculated by nonlinear regression according to the Michaelis-Menten equation. Alarmone hydrolysis was measured from hydrolysis of ^32^P-labeled alarmones, while NADPH hydrolysis was measured from inorganic phosphate release using a malachite green assay. *Xcc*SAH was used at 25 nM for pppApp, 50 nM for (pp)pGpp, and 1 μM for NAPDH. Each value represents the mean and standard deviation from three independent experiments.

In addition to the alarmones, we tested whether *Xcc*SAH hydrolyzes NADPH (32), the substrate of the metazoan SAH Mesh1 (28). Since *Xcc*SAH lacks a known nicotinamide riboside interaction (NR) motif (Fig. 1B), we were unsure whether *Xcc*SAH would be able to hydrolyze NADPH. Therefore, we incubated NADPH with or without *Xcc*SAH *in vitro*, followed by chemical validation of the product in the reaction mix by using liquid chromatography-mass spectrometry (LC-MS). Interestingly, we detected significant (~80%) hydrolysis of NADPH to NADH in the presence of *Xcc*SAH, while no NADH generation was detected in the enzyme-free control (Fig. 3A and B). This finding demonstrated that *Xcc*SAH is also an NADPH phosphatase. Next, we measured the catalytic parameters of *Xcc*SAH on NADPH hydrolysis by monitoring inorganic phosphate release in a malachite green assay (Fig. 3C and D). *Xcc*SAH hydrolyzed NADPH with a *K_m_* of 132 ± 39.1 μM and catalytic efficiency (*k*_cat_/*K_m_*) of (3.63 ± 0.30) × 10^2^ s^−1^ M^−1^ (Fig. 3E). This catalytic efficiency was ~40-fold lower than that reported for Mesh1 (32), in agreement with the lack of an optimized NR motif. Together, these results indicate that *Xcc*SAH is a promiscuous enzyme which can hydrolyze different alarmones and NADPH.

**FIG 3:**
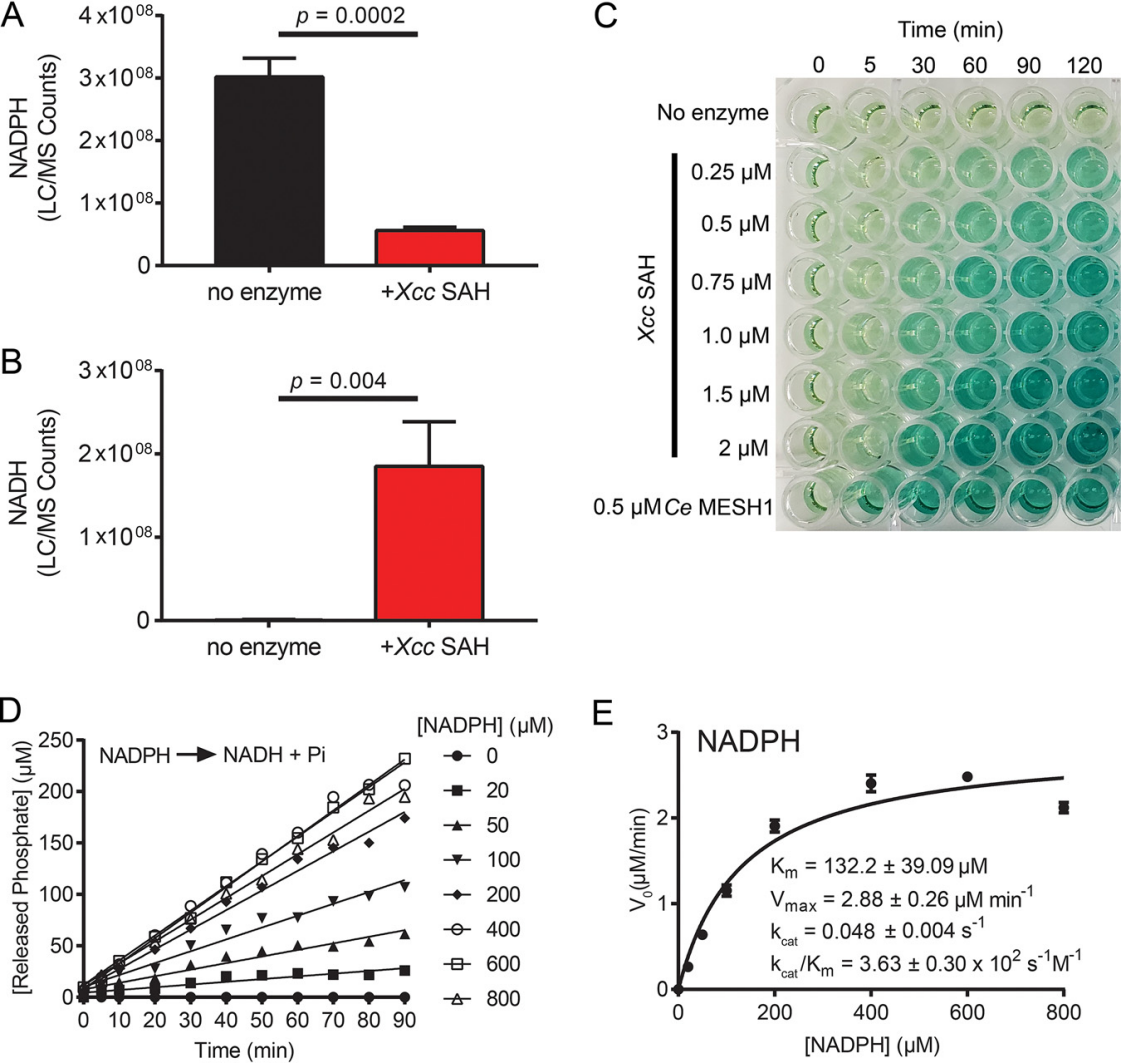
*Xcc*SAH is an NADPH phosphatase *in vitro*. (A and B) *Xcc*SAH dephosphorylates NADPH *in vitro*. Reaction was carried out at 37°C in 20 mM Tris-HCl (pH 8), 1 mM MnCl_2_, and 100 μM NADPH with or without 1 μM *Xcc*SAH for 60 min. Samples were quenched with 0.5 M formic acid and subjected to LC-MS validation and quantitation of NADPH (A) and NADH (B). (C) Detection of inorganic free phosphate released from NADPH dephosphorylation by *Xcc*SAH. NADPH hydrolysis reaction was carried out at 37°C in 20 mM Tris-HCl (pH 8), 1 mM MnCl_2_, 1 mM NADPH, and different concentrations of *Xcc*SAH. A 0.5 μM concentration of C. elegans Mesh1 (*Ce* Mesh1) was used as a positive control. Reaction aliquots were quenched with 0.5 M formic acid at different times as indicated and subjected to malachite green assay for free phosphate quantitation. The formation of malachite green-phosphate complex was proportional to the release of free phosphate from NADPH dephosphorylation. (D and E) Kinetics of NADPH hydrolysis by *Xcc*SAH. (D) Hydrolysis reaction was carried out as above with 1 μM *Xcc*SAH and different concentrations of NADPH. Rate of phosphate release was measured using a malachite green assay. (E) Hydrolysis kinetics of NADPH by *Xcc*SAH from the data in panel D. Data were fitted by nonlinear regression to the Michaelis-Menten equation. Error bars indicate standard deviations from three independent experiments. *P* values were determined using Student's *t* test.

### *Xcc*SAH reduces pGpp, ppGpp, pppGpp, and NADPH levels in X. campestris pv. *campestris* during starvation.

**FIG 4:**
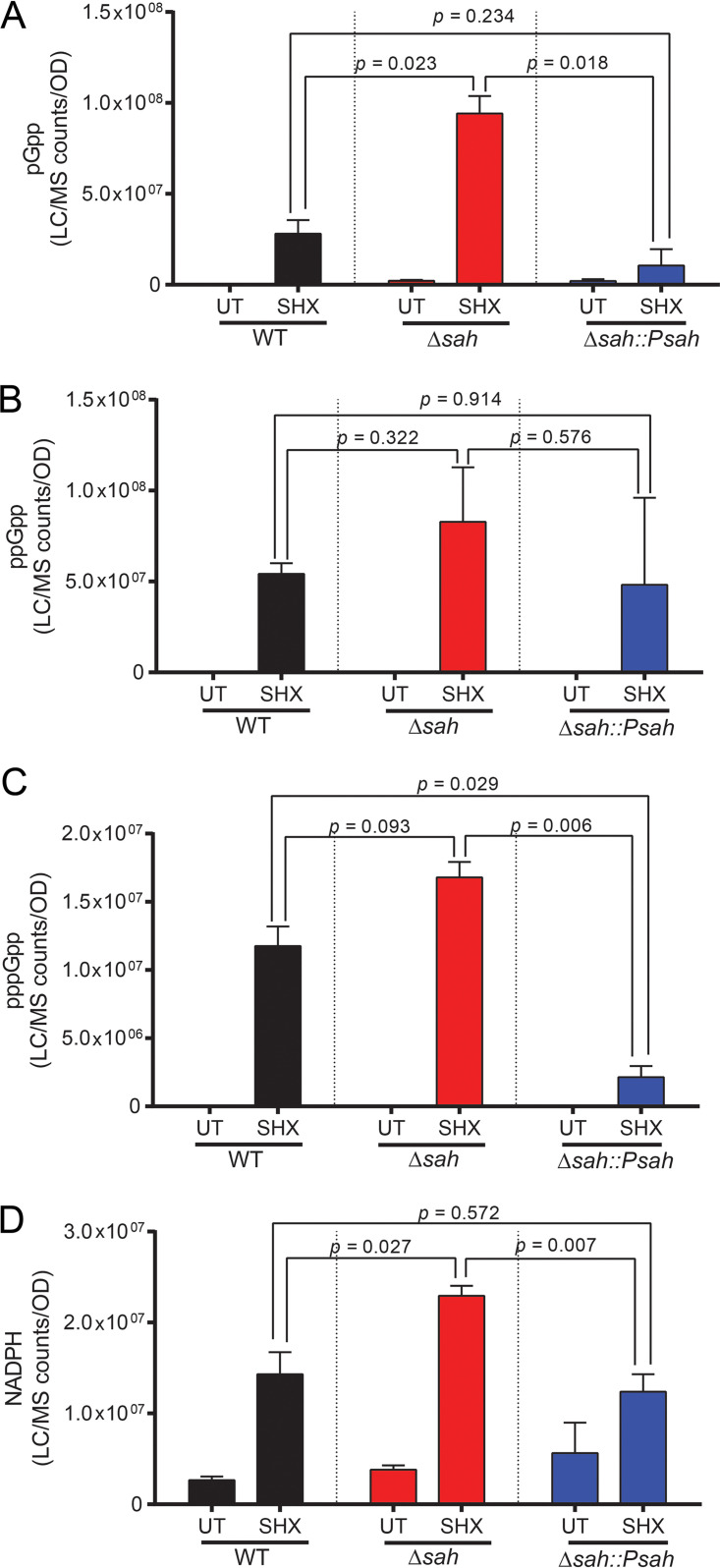
*Xcc*SAH reduces (pp)pGpp and NADPH levels in X. campestris pv. *campestris* during amino acid starvation. Levels of pGpp (A), ppGpp (B), pppGpp (C), and NADPH (D) in X. campestris pv. *campestris* WT, Δ*sah*, and Δ*sah*::P*sah* strains before and after amino acid starvation. Cultures were harvested before (UT) and after 30 min serine hydroxamate (SHX) treatment. Alarmones and NADPH were quantitated from extracted cellular metabolites using LC-MS. Normalized ion count represents LC-MS ion count per OD_600_ per unit volume of the culture. Error bars indicate standard errors from two independent experiments. *P* values were determined using Student's *t* test.

Since *Xcc*SAH can hydrolyze different alarmones and NADPH *in vitro*, next we studied whether it affects their levels *in vivo.* The guanylate alarmones pppGpp and ppGpp are induced by amino acid starvation in a variety of bacterial species (3). In the Gram-positive soil bacterium Bacillus subtilis, pGpp also accumulates to high levels during amino acid starvation through hydrolysis of (p)ppGpp (12). We measured the levels of pGpp, ppGpp, pppGpp, and NADPH using LC-MS (25) in wild-type X. campestris pv. *campestris*, Δ*sah*, and Δ*sah*::P*sah* complementation strains before and after treatment with a nonfunctional serine analog, serine hydroxamate (SHX), which mimics amino acid starvation in bacteria. In wild-type X. campestris pv. *campestris*, we detected increases of pGpp, ppGpp, pppGpp, and NADPH upon 30 min of SHX treatment (Fig. 4A to D), while no (p)ppApp was detected. The presence of pGpp suggests that X. campestris pv. *campestris* RSHs may be able to produce pGpp, as recently reported in Clostridiodes difficile (19), or that it contains Nudix hydrolases that can convert (p)ppGpp to pGpp, similar to NahA in B. subtilis (12). Importantly, the Δ*sah* mutant showed elevation of pGpp (Fig. 4A), ppGpp (Fig. 4B), pppGpp (Fig. 4C), and NADPH (Fig. 4D) compared to the wild type (WT) or the Δ*sah*::P*sah* complemented strain. These results support the hypothesis that *Xcc*SAH can hydrolyze both alarmone and NADPH in X. campestris pv. *campestris* cells during starvation.

### *Xcc*SAH has no detectable effect on plant pathogenesis, biofilm formation, or survival in soil.

**FIG 5:**
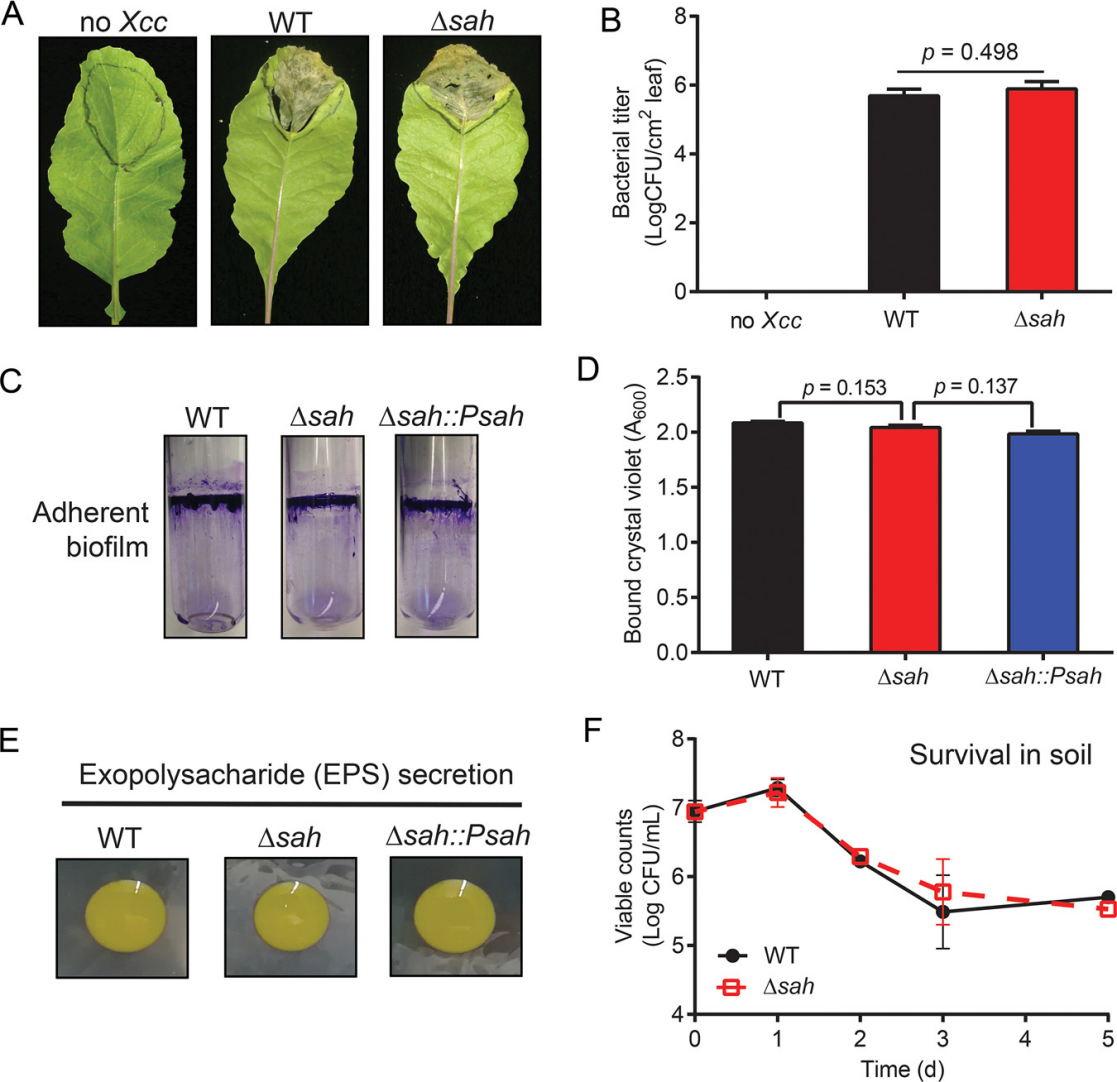
*Xcc*SAH had an undetectable effect on plant pathogenicity or biofilm formation in X. campestris pv. *campestris*. (A and B) Leaf infection by X. campestris pv. *campestris* WT and Δ*sah*. (A) Visual symptoms of disease on radish (Raphanus sativus L. cv. Japan 501) leaves inoculated with X. campestris pv. *campestris* suspensions at 7 dpi. N indicates the negative control (0.85% NaCl). Six individual seedlings were used for each experiment, and the entire experiment was conducted twice. (B) Bacterial titers recovered from inoculated radish leaves at 7 dpi. (C to E) Biofilm formation of X. campestris pv. *campestris* WT, Δ*sah*, and Δ*sah*::P*sah*. (C) Biofilm formation of the X. campestris pv. *campestris* strains on glass tubes. The glass-bound biofilm was stained with crystal violet. (D) Biofilm quantitated from data in panel C after dissolving in absolute ethanol. (E) Exopolysaccharide (EPS) secretion of the X. campestris pv. *campestris* strains after 4 days of incubation on LBA containing 2% sucrose. (F) Survival of X. campestris pv. *campestris* WT and Δ*sah* in soil planted with Chinese cabbage. Error bars indicate standard deviations from three independent experiments. *P* values were determined using Student's *t* test.

Since X. campestris pv. *campestris* is a plant pathogen, we tested whether *Xcc*SAH was associated with pathogenicity in plants by infecting radish leaves with X. campestris pv. *campestris* wild type or Δ*sah* mutant (Fig. 5A and B). No detectable differences were observed for the visual symptoms (Fig. 5A) or bacterial titer postinfection (Fig. 5B) in the leaves infected by either wild type or Δ*sah* mutant. Next, we evaluated potential roles of *Xcc*SAH on biofilm formation (Fig. 5C and D) and exopolysaccharide (EPS) secretion (Fig. 5E) and found no significant differences. In addition, we monitored the survival of X. campestris pv. *campestris* wild type and Δ*sah* mutant in a cabbage-inhabited soil over time and found no detectable difference in viability (Fig. 5F). Collectively, the results suggested that *Xcc*SAH is dispensable for plant pathogenesis and soil survival in X. campestris pv. *campestris*.

### *Xcc*SAH contributes to survival against competing bacteria and growth in defined media supplemented with amino acids.

**FIG 6:**
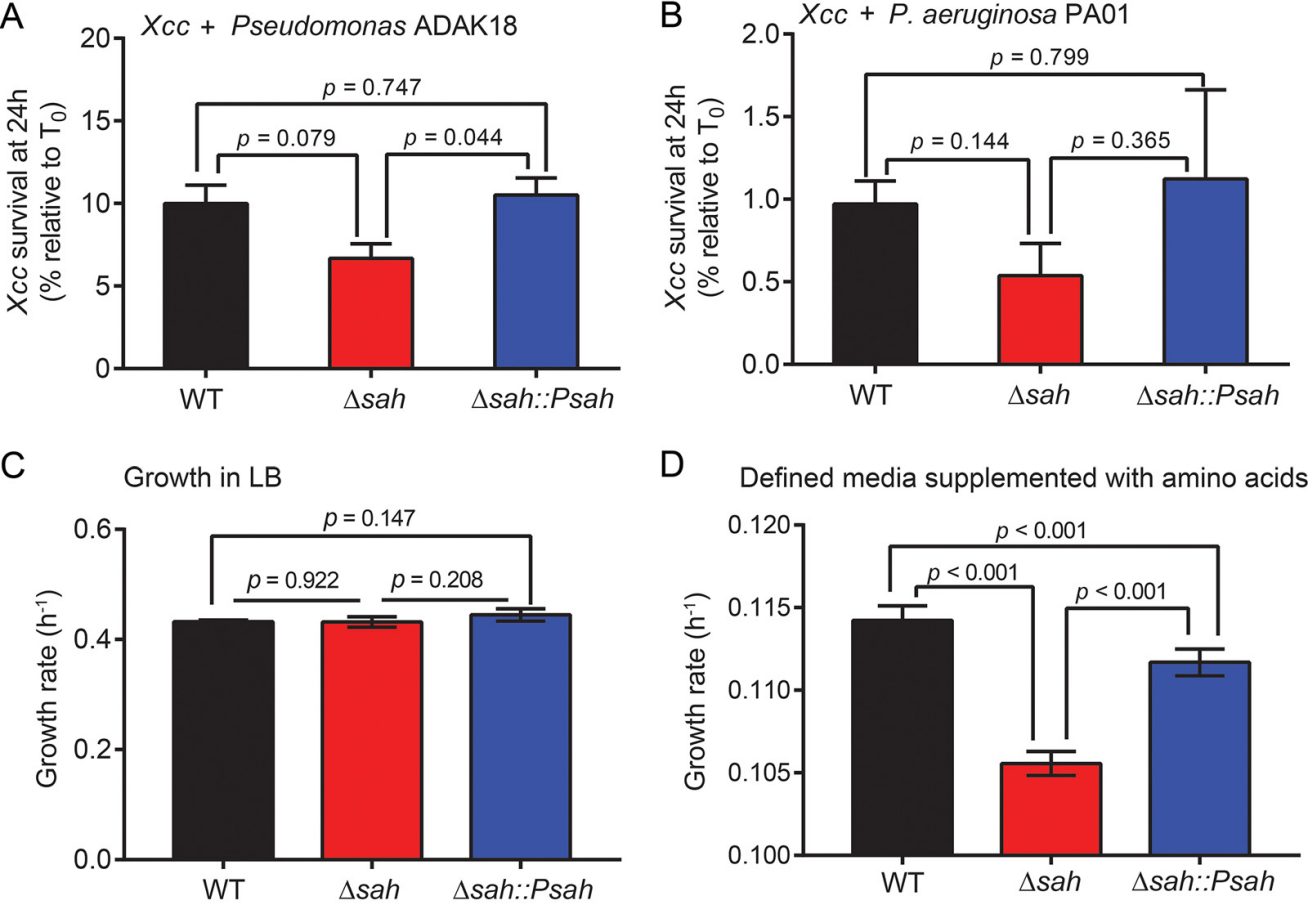
*Xcc*SAH contribute to X. campestris pv. *campestris* survival against bacterial competitors, as well as growth in defined media supplemented with amino acids. (A and B) *Xcc*SAH contributes to survival against Pseudomonas. X. campestris pv. *campestris* WT, Δ*sah*, or Δ*sah*::P*sah* cultures were mixed with cultures of Pseudomonas ADAK18 (Tas1-carrying strain) (A) or Pseudomonas aeruginosa PA01 (strain without Tas1) (B) and patched on LB agar. Bacteria on patches were recovered before and after 24 h of incubation. Survival of X. campestris pv. *campestris* strains was measured by plating on X. campestris pv. *campestris*-selective plates followed by colony counting. (C and D) *Xcc*SAH contributes to growth in defined media supplemented with amino acids. X. campestris pv. *campestris* WT, Δ*sah*, or Δ*sah*::P*sah* cells were measured for their doubling times in either LB (C) or defined media supplemented with amino acids (D). Error bars indicate standard errors from three independent experiments. *P* values were determined using Student's *t* test.

As *Xcc*SAH hydrolyzes pppApp most efficiently, we speculated that it may promote competitive fitness of X. campestris pv. *campestris* against (p)ppApp synthesis by the interbacterial toxin Tas1 from Pseudomonas during competition (29). To test this, we measured the survival of X. campestris pv. *campestris* cocultured with *tas1*-containing Pseudomonas ADAK18, which is a soil bacterium (45) (see Fig. S1 in the supplemental material) that shares similar habitat as X. campestris pv. *campestris*, or Pseudomonas aeruginosa PA01, which does not carry Tas1 (14). In both cases, coculture with Pseudomonas led to a strong reduction in the number of viable X. campestris pv. *campestris* cells down to 1% to 10%, suggesting that Pseudomonas can effectively kill X. campestris pv. *campestris* cells. X. campestris pv. *campestris* cells lacking *sah* displayed ~40 to 50% reduced survival compared to wild type or the complementation strains (Fig. 6A and B), suggesting that *Xcc*SAH promotes survival against competition with Pseudomonas with or without the Tas1 toxin.

10.1128/mbio.02422-22.1 FIG S1 Homology of Tas1 in Pseudomonas aeruginosa PA14 and Pseudomonas ADAK18. Tas1 in PA14 and ADAK8 are highly similar, with an identity of 74.38% (Clustal Omega). The red triangles indicate key catalytic residues of Tas1 in PA14 (14). Download FIG S1, TIF file, 1.8 MB. Copyright © 2022 Fung et al. 2022 Fung et al. https://creativecommons.org/licenses/by/4.0/ This content is distributed under the terms of the Creative Commons Attribution 4.0 International license.

Finally, we quantified the potential impact of *sah* during steady-state planktonic growth. X. campestris pv. *campestris* lacking *sah* grew similarly to wild-type cells in Luria-Bertani (LB) medium (Fig. 6C) but displayed compromised growth in morpholinopropanesulfonic acid (MOPS) defined medium supplemented with amino acids, with an ≈10% reduction in growth rate (Fig. 6D) and no detectable difference in the optical density/viability ratio (Fig. S2). The growth reduction was complemented by ectopic *sah* expression (Fig. 6D), suggesting that the defect is dependent on *Xcc*SAH. These results suggest that *Xcc*SAH modestly promotes growth of X. campestris pv. *campestris*, which will provide a selective advantage over the long run.

10.1128/mbio.02422-22.2 FIG S2 Growth reduction of *sah* mutant is not due to difference in optical densities. X. campestris pv. *campestris* WT, Δ*sah*, or Δ*sah*::P*sah* cells grown in defined media supplemented with amino acids were measured for their CFU per OD_600_ unit. Error bars indicate standard errors from three independent experiments. *P* values were determined using Student’s *t* test. Download FIG S2, TIF file, 0.8 MB. Copyright © 2022 Fung et al. 2022 Fung et al. https://creativecommons.org/licenses/by/4.0/ This content is distributed under the terms of the Creative Commons Attribution 4.0 International license.

### *Xcc*SAH strongly impacts cellular NADH levels in X. campestris pv. *campestris* during growth in defined media supplemented with amino acids.

**FIG 7:**
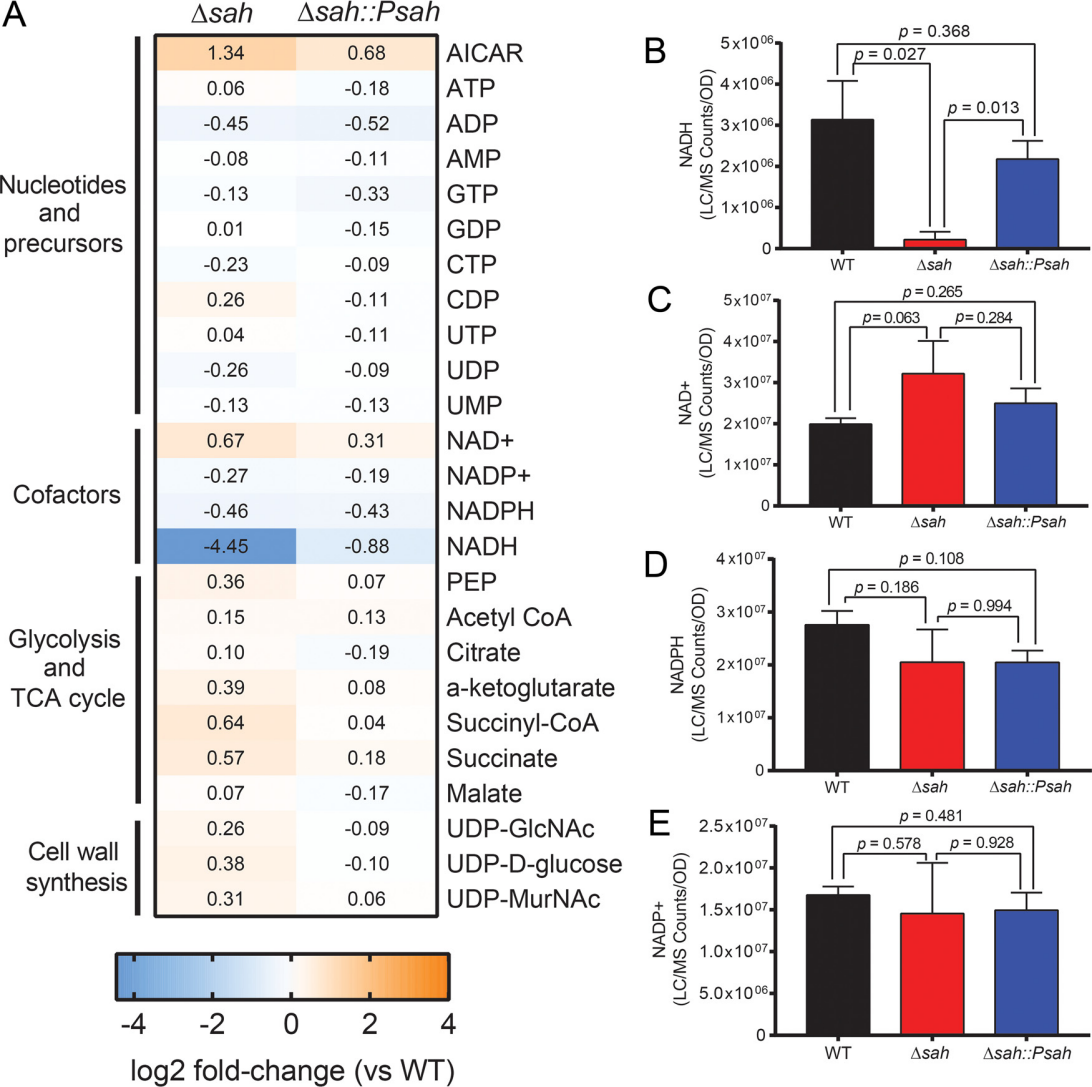
*Xcc*SAH regulates NADH levels in X. campestris pv. *campestris* during growth in defined media supplemented with amino acids. (A) Heat map of metabolite changes in Δ*sah* or Δ*sah*::P*sah* strains relative to WT. Cells were grown in defined media supplemented with amino acids and harvested at exponential phase for metabolite extraction and LC-MS analysis. Values shown are mean relative changes of OD_600_-normalized ion counts (log_2_). PEP, phosphoenolpyruvate; UDP-GlcNAc, UDP-*N*-acetylglucosamine; UDP-MurNAc, UDP-*N*-acetylmuramate. (B to E) NADH (B), NAD^+^ (C), NADPH (D), and NADP^+^ (E) levels in X. campestris pv. *campestris* WT, Δ*sah*, and Δ*sah*::*Psah* strains during exponential growth in defined media supplemented with amino acids. Ion counts were normalized to OD_600_. Error bars indicate standard errors from three independent experiments. *P* values were determined using Student's *t* test.

The modest growth defect we observed in amino acid-supplemented defined media suggests that *Xcc*SAH may impact cellular metabolism under conditions where (p)ppGpp alarmones are uninduced. To test this hypothesis, we performed metabolite profiling of wild-type, Δ*sah*, and *sah* complementation strains in the same media (Fig. 6D) using LC-MS. Comparison of metabolite levels between wild type and Δ*sah* mutants revealed that the levels of most metabolites, including nucleotides and cell wall precursors, were largely similar between the strains (Fig. 7A). In addition, no (pp)pGpp or pppApp was detected in either WT or Δ*sah*, indicating that the alarmones were not induced or were below the detection limit during exponential growth under this condition. However, the level of the NADH cofactor was significantly lower (~10-fold) in the Δ*sah* mutant (Fig. 7B). In addition, the Δ*sah* mutant also displayed a small (~50%) increase in NAD^+^ compared to wild-type cells (Fig. 7C), while NADPH and NADP^+^ levels had no significant differences (Fig. 7D and E). The drop in NADH in the Δ*sah* mutant was not due to defects in the tricarboxylic acid (TCA) cycle, a major source of NADH generation, since the levels of TCA cycle metabolites remained similar or even slightly higher in the mutant (Fig. S3). Instead, the depletion of NADH due to loss of *Xcc*SAH corroborated our finding that *Xcc*SAH can generate NADH from NADPH *in vitro* (Fig. 3A and B). Importantly, reintroducing the *sah* gene to the Δ*sah* mutant restored NADH level back to wild-type levels (Fig. 7A and B). Together, the results suggest that *Xcc*SAH contributes to the maintenance of the NADH pool through dephosphorylation of NADPH.

10.1128/mbio.02422-22.3 FIG S3 Levels of TCA cycle metabolites in X. campestris pv. *campestris* WT, Δ*sah*, and Δ*sah*::P*sah* in defined media supplemented with amino acids. Levels of citrate (A), α-ketoglutarate (B), succinyl-CoA (C), succinate (D), malate (E), and oxaloacetate (F) in X. campestris pv. *campestris* WT, Δ*sah*, and Δ*sah*::P*sah* strains, as described and shown in Fig. 6A. Cells were grown in defined media supplemented with amino acids and harvested at exponential phase for metabolite extraction and LC-MS analysis. Ion counts were normalized to the OD_600_. Error bars indicate standard errors from three independent experiments. *P* values were determined using Student’s *t* test. Download FIG S3, TIF file, 1.2 MB. Copyright © 2022 Fung et al. 2022 Fung et al. https://creativecommons.org/licenses/by/4.0/ This content is distributed under the terms of the Creative Commons Attribution 4.0 International license.

## DISCUSSION

Small alarmone hydrolases have been identified across domains of life and play multiple roles in metazoans in response to environmental stresses. However, the physiological roles of small alarmone hydrolases are poorly characterized in bacteria, except as antitoxins. In this study, we characterized an orphan small alarmone hydrolase in the plant-pathogenic bacterium X. campestris pv. *campestris*. We found that *Xcc*SAH hydrolyzed different alarmones and cofactors *in vitro* and played multiple roles *in vivo*. First, *Xcc*SAH contributed to hydrolysis of (pp)pGpp in X. campestris pv. *campestris* during amino acid starvation, demonstrating a biological role of SAH in alarmone turnover. Second, *Xcc*SAH improved survival against bacterial predators such as Pseudomonas species. Third, *Xcc*SAH hydrolyzed NADPH to release NADH *in vitro* and had a strong effect on cellular NADH levels *in vivo*. Together, our findings suggest that bacterial SAH can function as a “Swiss army knife” of alarmone and nucleotide regulation during growth or upon stresses.

The substrate promiscuity we observed with *Xcc*SAH was intriguing. *Xcc*SAH is able to mediate nucleophilic attack of the phosphate 2′ or 3′ of ribose (3′ for alarmones and 2′ for NADPH), regardless of the length of the 5′ phosphates (mono-, bi-, or triphosphate) or the identity of the purine base (A or G). *Xcc*SAH belongs to the subgroup Mesh1-L (16), which is closely related to subgroup Mesh1, the metazoan SAH with promiscuity to remove the phosphate groups at 3′ for (p)ppGpp and 2′ for NADPH in the ribose ring (32), regardless of the identity of the purine base (A or G) (30). Other SAH homologs, such as Methylobacterium extorquens SAH (30), have been reported to display a more restricted substrate spectrum, hydrolyzing (p)ppApp but not (p)ppGpp. The different substrate spectrum in *Xcc*SAH could be associated with the distinct sequence features outside of the highly conserved catalytic residues essential for metal coordination and hydrolysis (Fig. 1B). First, *Xcc*SAH lacks a well-defined nicotinamide riboside (NR) interaction motif found in Mesh1. This could explain why *Xcc*SAH is as efficient against alarmones as Mesh1 (30), but its efficiency of NADPH hydrolysis is ~40 times less efficient than Mesh1 (32) (~3.63 × 10^2^ s^−1^ M^−1^ versus ~14.4 × 10^3^ s^−1^ M^−1^). Second, while *Xcc*SAH can hydrolyze the toxic alarmone pppApp, similar to Pseudomonas aeruginosa SAH (29) and M. extorquens SAH (30), it lacks the multiple prolines adjacent to the hydrolase domain (HD) motifs HD5 and -6 (Fig. 1B) proposed to increase substrate promiscuity (29). Interestingly, *Xcc*SAH uniquely contains a stretch of three glycines adjacent to the conserved hydrolase domain motifs HD3 and HD4 (Fig. 1B), which may also influence substrate specificity. Given the recent report that subtle structural variations between SAHs can lead to marked differences in hydrolysis properties (46), it is possible that *Xcc*SAH may have evolved unique features to achieve different degrees of substrate promiscuity and hydrolysis activities.

Apart from an increased substrate spectrum, the biochemical properties of *Xcc*SAH toward different substrates also differed (Table 3). *Xcc*SAH had higher catalytic efficiency for the alarmones than for NADPH, likely due to the difference between 3′-phosphates in the alarmones versus 2′-phosphates in NADPH (32). For alarmones, *Xcc*SAH had higher affinity for (pp)pGpp than pppApp, while catalytic activity was the greatest for pppApp, followed by pppGpp, ppGpp, and pGpp (Table 3). This suggests that *Xcc*SAH prefers alarmone substrates with an adenine over guanine nucleobase in addition to a higher number of 5′-phosphates. Physiologically, the different affinities and catalytic activities to substrates could lead to competition for enzyme activity when multiple substrates are present in the cell. The high catalytic efficiency for pppApp by *Xcc*SAH likely prioritizes the enzyme toward hydrolysis against toxic (p)ppApp accumulation from interbacterial attack, since hydrolases such as SpoT have no activity against (p)ppApp. The moderate (pp)pGpp hydrolysis efficiency by *Xcc*SAH can enable the enzyme to assist (pp)pGpp turnover during stress when the cells have high (pp)pGpp. The lower efficiency of NAPDH hydrolysis suggests that NADPH/NADH regulation by *Xcc*SAH is more prominent when the levels of competing alarmone substrates are low and cofactor levels are high, such as during unstressed growth. Thus, *Xcc*SAH provides diverse regulation of alarmones and cofactors, according to their relative concentrations in the cell. In addition, gene expression control or interacting regulators may also be present to further modulate *Xcc*SAH level and activity.

Our finding that *Xcc*SAH is an NADPH phosphatase suggests that NADPH hydrolysis is an evolutionarily conserved function of both bacterial and metazoan SAH and Mesh1. Based on our *in vitro* data, *Xcc*SAH has a *K_m_* of ~130 μM to NADPH. This is physiologically relevant to our estimated NADPH concentration of ~570 μM in X. campestris pv. *campestris* grown in amino acid-defined media (Table 4). In comparison, NADH concentration was ~87 μM (Table 4), which was similar to that in exponentially growing E. coli (47). The difference between cellular NADPH and NADH levels in X. campestris pv. *campestris* suggests that *Xcc*SAH can potentially release NADH from NADPH to facilitate growth under suboptimal nutrient conditions. This possibility is supported by our observation that the Δ*sah* mutant had reduced NADH and growth, both of which were restored by reintroduction of the *sah* gene. Compared to metazoan Mesh1, which controls NADPH levels to regulate NADPH-driven pathways such as ferroptosis (32), *Xcc*SAH appears to play a role in NADH regeneration from the NADPH pool.

**TABLE 4:** Estimated NAD(H) and NADP(H) concentrations in X. campestris pv. *campestris* wild type and *sah* mutants grown in defined media supplemented with amino acids^*a*^

Metabolite	Cellular concn in X. campestris pv. *campestris* strain (μM)		
	WT	Δ*sah*	Δ*sah*::P*sah*
NAD^+^	416.17 ± 31.03	673.72 ± 95.83	522.41 ± 75.88
NADP^+^	350.68 ± 21.50	304.49 ± 73.35	312.69 ± 44.09
NADPH	577.31 ± 54.81	429.24 ± 74.85	428.51 ± 46.89
NADH	87.15 ± 24.35	4.61 ± 2.27	31.90 ± 15.60

a Estimations are based on LC-MS data presented in Fig. 7. Each value represents the mean and standard error from three independent experiments.

As a plant pathogen, X. campestris pv. *campestris* is exposed to both plant-associated and nonplant habitats (48) that can be subjected to diverse environmental changes, such as nutrient fluctuations and attack and competitions from other microorganisms. To facilitate adaptation, *Xanthomonas* employs multiple signaling systems to regulate stress responses (49), including the nucleotide second messenger c-di-GMP, which affects virulence and motility (50), and the nucleotide alarmone (p)ppGpp, produced by RelA and SpoT and which is required for virulence in X. campestris pv. *campestris* (41). In contrast, we were not able to detect an essential role of *Xcc*SAH in X. campestris pv. *campestris* pathogenesis; instead, we revealed that *Xcc*SAH contributes to interspecies competition, growth fitness, and NADH metabolism in X. campestris pv. *campestris*. Thus, it is possible that *Xcc*SAH functions as a tuner of stress response and metabolism with biological roles diversified from RelA and SpoT. Given the widespread presence of SAHs in bacteria, it is plausible that this differential role is conserved in other *Xanthomonas* species.

## MATERIALS AND METHODS

### Bioinformatic analysis.

The putative Xanthomonas campestris pv. *campestris relA* and *spoT* sequences identified in a previous report of the RelA/SpoT superfamily (16) were retrieved from the X. campestris pv. *campestris* 8004 genome database (NCBI). Functional domains within RelA and SpoT were identified using the InterPro online tool (https://www.ebi.ac.uk/interpro/search/sequence/). Amino acid sequences of both the RelA and SpoT hydrolase domains were used as a query to search the X. campestris pv. *campestris* 8004 genome for other (p)ppGpp hydrolase homologs. For comparative analysis of hydrolase domains (HDs) in SAHs from different organisms, amino acid sequences were retrieved from NCBI and analyzed using Clustal Omega (https://www.ebi.ac.uk/Tools/msa/clustalo/) and Jalview (51).

### Bacterial strains, plasmids, and culture conditions.

The bacterial strains and plasmids used in this study are listed in (Table 5, and primers are listed in Table 6. X. campestris pv. *campestris* 8004 (52) and its isogenic derivatives were cultured at 28°C with shaking (250 rpm) in LB broth (5 g/liter yeast extract, 10 g/liter NaCl, and 10 g/liter tryptone), or defined medium supplemented with amino acids (MOPS EZ defined medium [Teknova] containing all amino acids, 0.2% glucose replaced by 0.2% glutamate, and without 10× ACGU [a solution of adenine, cytosine, guanine, and uracil]). Escherichia coli strains were cultured in LB broth at 37°C with shaking (250 rpm). When required, solid medium was prepared by the addition of 15 g/liter agar, and selective antibiotics were added at the following concentrations: rifampin, 50 μg/mL; kanamycin, 50 μg/mL; chloramphenicol, 20 μg/mL; carbenicillin, 100 μg/mL; and tetracycline, 5 μg/mL for X. campestris pv. *campestris* and 15 μg/mL for E. coli.

**TABLE 5:** Bacterial strains and plasmids used in this study

Strain or plasmid	Description^*a*^	Source
Bacterial strains		
JDW4203	X. campestris pv. *campestris* wild-type isolate 8004, Rif^r^	52
JDW4204	X. campestris pv. *campestris* 8004 Δ*sah*, Rif^r^	This study
JDW4205	X. campestris pv. *campestris* 8004 Δ*sah* pLAFR3-*sah*, Rif^r^ Tc^r^	This study
JDW4209	E. coli BL21(DE3) harboring pLIC-*sah* plasmid, Amp^r^	This study
JDW3005	P. aeruginosa PA01	Lab collection
JDW4208	Pseudomonas sp. ADAK18	45
Plasmids		
p2P24Km	Suicide vector for in-frame deletion, derived from pEx18-KCL containing SacB selectable marker, Km^r^	53
p2P24Km-*sah*	p2P24Km containing SAH fragment, Km^r^	This study
pRK600	Helper plasmid for triparental mating ColE1 *oriV tra*^+^ RP4 *oriT*, Cm^r^	54
pLAFR3	X. campestris pv. *campestris* expression vector containing RK2 replicon, Tc^R^	56
pLAFR3-*sah*	Complementation vector, pLAFR3 containing *sah* gene, Tc^r^	This study
pLICtrPC-HA	Protein expression vector, Amp^r^	Lab stock
pLIC-*sah*	Protein expression vector, pLICtrPC-HA containing *Xcc*SAH gene, Amp^r^	This study
pLIC-Mesh1	Protein expression vector, pLICtrPC-HA containing C. elegans Mesh1 gene, Amp^r^	This study

a Rif^r^, Km^r^, Cm^r^, Tc^r^ and Amp^r^ indicate resistance to rifampicin, kanamycin, chloramphenicol, tetracycline and ampicillin, respectively.

**TABLE 6:** Oligonucleotides used in this study

Oligo	Sequence (5′→3′)^*a*^
oJW3946	TACTTCCAATCCAATGCAATGACTGCGTTAACGGAGCGCTA
oJW3947	TTATCCACTTCCAATGTTATTACGCTACAGCATGAGCCGGTAC
oJW1124	CCGCACCTGTGGCGCCGGTG
oJW492	GCTTTGTTAGCAGCCGGATCAG
DLH120	CCGTAGCACTTAGTGCAATG
DLH125	GCATTTCCATCGGTCACGATTG
oJW4314	ATGACCATGATTACGTCTCCTGCCCCGGTGGGT
oJW4315	CAGTGATGCGCGCGGCCGGCGCAGCGGTGCAAT
oJW4316	GGGGCGTCCAATCCTGAAGATGGCCGCAGTATC
oJW4317	TGCATGCCTGCAGGTATGATGCTGTGCAGCGGC
oJW4318	CGGAATTCCTGCCCCGGTGGGTACT (EcoRI)
oJW4319	CCAAGCTTGCGGCCACTGGTAGTGC (HindIII)
oJW3991	CAATACCGAGCGTGGTGAGG
oJW3992	GGCGGGCGAACAAATAGC
oJW4030	CGGGATCCGCTCCTGCTCGCTGACTTCG (BamHI)
oJW4031	CCAAGCTTCCGATCCACCGTGATTGCA (HindIII)
oJW4032	TGCCGTGCTCGTGTTCGGGGG
oJW4033	GAGTTAGCTCACTCATTAGG

a Underlined nucleotides indicate the location of restriction sites used for cloning (restriction enzymes are shown in parentheses).

The X. campestris pv. *campestris* Δ*sah* mutant was generated using the triparental mating method. Fragments 500 bp upstream and downstream of the *sah* gene were amplified using primers oJW4314/oJW4315 and oJW4316/oJW4317, respectively. The resulting PCR products were ligated with primers oJW4318/oJW4319 using the In-Fusion method, followed by EcoRI/HindIII digestion and ligation into p2P24Km vector (53) to generate the donor vector p2P24Km-*sah*. Triparental mating was conducted using E. coli donors containing p2P24Km-*sah* with an E. coli helper strain containing pRK600 (54) to transform X. campestris pv. *campestris* 8004 (WT), followed by double selection with rifampin and kanamycin. Xanthomonad-like colonies (yellow in color) were picked and verified by PCR using X. campestris pv. *campestris*-specific primers DLH120/DLH125 (55) and X. campestris pv. *campestris sah*-specific primers oJW3991/oJW3992.

Complementation of X. campestris pv. *campestris* strains ectopically expressing *sah* in the Δ*sah* background were also generated by triparental mating. The full-length *sah* gene including the native promoter was amplified using the oJW4030/oJW4031 primer set and cloned into the pLAFR3 (56) vector to generate the plasmid pLAFR3-*sah*. Triparental mating was conducted using E. coli donors containing pLAFR3-*sah* with an E. coli helper strain containing pRK600 (54) to transform X. campestris pv. *campestris* Δ*sah*, followed by double selection with rifampin and tetracycline.

To generate pLIC-*sah* for expression and purification of *Xcc*SAH protein, the *sah* coding sequence was amplified by PCR using primers oJW3946/oJW394 and cloned into pLICtrPC-HA (a His tag overexpression vector) using ligation-independent cloning (57, 58). The resulting plasmid was transformed into E. coli BL21(DE3) for protein expression.

### Expression and purification of *Xcc*SAH and C. elegans Mesh1.

His-tagged *Xcc*SAH or C. elegans Mesh1 was overexpressed in E. coli BL21(DE3) containing the expression plasmid pLIC-*sah* or pLIC-Mesh1, respectively. Cells were grown in LB with 100 μg/mL carbenicillin at 28°C overnight, diluted 1:50 in fresh LB with 100 μg/mL carbenicillin, and grown at 37°C with shaking to an optical density at 600 nm (OD_600_) of ~0.7. Expression was induced by adding 1 mM isopropyl-β-d-thiogalactopyranoside at 37°C for 4 h. Cells were pelleted at 5,000 × *g* for 10 min at 4°C and stored at −80°C. Cell pellets were resuspended in lysis buffer (50 mM NaH_2_PO_4_ [pH 8], 300 mM NaCl, 10 mM imidazole, 20 mg/mL lysozyme [Sigma]) and treated with benzonase endonuclease (Fisher Scientific) for 30 min on ice. Cell lysate was centrifuged at 12,000 × *g* 4°C for 15 min to collect the supernatant. The supernatant was loaded into a Ni-nitrilotriacetic acid spin column (Qiagen) by centrifugation at 850 × *g* at 4°C for 5 min, followed by washing twice with wash buffer (50 mM NaH_2_PO_4_ [pH 8], 300 mM NaCl, and 20 mM imidazole). Protein was eluted with 600 μL elution buffer (50 mM NaH_2_PO_4_ [pH 8], 300 mM NaCl, and 500 mM imidazole) and dialyzed twice with dialysis buffer (20 mM HEPES [pH 7.5], 250 mM NaCl, 1 mM dithiothreitol [DTT], and 10% glycerol [vol/vol]) at 4°C. The purified protein was concentrated and analyzed using SDS-PAGE, and the protein concentration was measured by the Bradford assay (Bio-Rad).

### *In vitro* synthesis of (p)ppGpp, pGpp and pppApp.

pppGpp was synthesized from 8 mM ATP and 6 mM GTP using RelSeq_1-385_ (59) in a buffer containing 25 mM bis-Tris propane (pH 9.0), 15 mM MgCl_2_, and 0.5 mM DTT at 37°C for 6 h. For production of [5′-α-^32^P]pppGpp, [α-^32^P]GTP was used instead of nonradiolabeled GTP. ppGpp and pGpp were synthesized from pppGpp using GppA (59) and NahA (12), respectively, as described previously (59). pppApp was kindly shared by Boyuan Wang and Michael Laub. [5′-γ-^32^P, 3′-β-^32^P]pppApp synthesis was performed by incubating [γ-^32^P]ATP with 10 μM Staphylococcus aureus SasA (29) in 10 mM HEPES, 10 mM MgCl_2_, 10 mM KCl, and 100 mM NaCl (pH 7.5) at 37°C overnight. The synthesis reaction was quenched using 2.4 volumes of CHCl_3_ followed by incubation at 95°C for 15 s. Purification of nucleotides was performed using anion-exchange column (HiTrap QFF 1 mL; GE Healthcare) using a gradient of buffer A (0.1 mM LiCl, 0.5 mM EDTA, 25 mM Tris-HCL; pH 7.5) and buffer B (1 M LiCl, 0.5 mM EDTA, 25 mM Tris-HCL; pH 7.5). Purity of synthesized nucleotides was determined by spotting on polyethyleneimine (PEI)-cellulose TLC plates (Millipore) and resolved in 1.5 M KH_2_PO_4_ at pH 3.4. TLC plates were exposed on storage phosphor screens (GE Healthcare) and scanned on a Typhoon imager (GE Healthcare).

### Alarmone hydrolysis assay.

Hydrolysis of (p)ppGpp, pGpp, and pppApp was carried out in a reaction mixture containing 20 mM Tris-HCl (pH 7.5), ~0.3 nM ^32^P-radiolabeled nucleotide (pGpp, ppGpp, pppGpp, or pppApp), 1 mM MnCl_2_, and 240 nM purified hydrolases (B. subtilis Rel, B. subtilis Rel^R44Q^ [a hydrolase-defective variant], or *Xcc*SAH) at 37°C. To compare the effect of magnesium versus manganese on hydrolysis, 1 mM MnCl_2_ was replaced with 10 mM MgCl_2_. At indicated times, 10 μL of reaction mixture was added to 2 μL of 2 M formic acid to quench the reaction. Two microliters of the quenched sample was spotted on PEI-cellulose TLC plates (Millipore) and resolved in 1.5 M KH_2_PO_4_ (pH 3.4) buffer. TLC plates were dried and exposed on storage phosphor screens (GE Healthcare) and scanned on a Typhoon imager (GE Healthcare). Identities of the hydrolysis products were verified with nucleotide standards run in parallel or by using high-performance LC-MS.

For kinetic assays of alarmone hydrolysis by *Xcc*SAH, the reaction mixture contained 20 mM Tris-HCl (pH 7.5), 1 mM MnCl_2_, 50 nM *Xcc*SAH, ~0.1 nM ^32^P-radiolabeled nucleotide, and the indicated concentrations of corresponding nonradioactive nucleotide. In the case of pppApp hydrolysis, *Xcc*SAH was used at 25 nM. The reaction was carried out at 37°C, and 10 μL of the reaction mixture was removed at 0 min, 0.5 min, 1 min, 1.5 min, and 2 min into 2 μL of 2 M formic acid to quench the reaction. Two microliters of the quenched sample was subjected to thin-layer chromatography. The levels of substrate and product were quantified using a Typhoon imager. *K_m_*, *V*_max_, and *k*_cat_ were determined by fitting to the Michaelis-Menten equation using Prism 7 (GraphPad).

### NADPH hydrolysis assay.

To determine the products of NADPH hydrolysis by *Xcc*SAH, 100 μM NADPH was incubated with or without 1 μM *Xcc*SAH in 20 mM Tris-HCl (pH 8) with 1 mM MnCl_2_ for 60 min at 37°C. The reaction was quenched with 0.5 M formic acid and diluted 10-fold with HPLC-grade H_2_O. The diluted reaction mixture was subjected to HPLC-MS analysis as described previously (12, 25).

Kinetics of NADPH hydrolysis by *Xcc*SAH were determined by measuring the rate of phosphate release using the malachite green phosphate assay kit (Sigma). The hydrolysis reaction was carried out at 37°C in 20 mM Tris-HCl (pH 8), 1 mM MnCl_2_, 1 mM NADPH, and different concentrations of *Xcc*SAH. As a positive control, purified C. elegans Mesh1 was used at 0.5 μM. For kinetic assays, reactions were carried out at 37°C in 20 mM Tris-HCl (pH 8), 1 mM MnCl_2_, 1 μM *Xcc*SAH, and increasing concentrations of NADPH. At indicated times, an aliquot of the reaction mixture was diluted into freshly prepared working reagent, mix gently, and incubated at room temperature for 30 min. Color development was measured by absorbance at 620 nm using the Synergy2 microplate reader (Biotek); concentration of released free phosphate was determined from the standard curve prepared as described in the manufacturer’s manual. Hydrolysis kinetics were determined by fitting the rate of phosphate release to the Michaelis-Menten equation using Prism 7 (GraphPad).

### Metabolite extraction and detection using HPLC-tandem MS.

For detection of metabolites during amino acid starvation, fresh colonies of X. campestris pv. *campestris* wild type, Δ*sah* mutant, and Δ*sah*::P*sah* complementation strain were inoculated into 5 mL LB broth and incubated at 28°C with shaking (200 rpm) overnight. Cells were harvested by centrifugation and resuspended at an OD_600_ of ≈0.3 in M9 broth (30 g/liter Na_2_HPO_4_, 15 g/liter KH_2_PO_4_, 2.5 g/liter NaCl, 5 g/liter NH_4_Cl, 2 mM MgSO_4_·7H_2_O, 0.1 mM CaCl_2_, and 0.2% glucose) containing 0.5 mg/mL serine hydroxamate (Sigma). Cultures were harvested at 0 min and 30 min after resuspension. Ten-milliliter cultures were filtered through a 0.45-μm polytetrafluoroethylene membrane (Sartorius) and then immediately transferred to 3 mL of prechilled 50:50 (vol/vol) chloroform-water, followed by vortexing to quench metabolism and to extract soluble metabolites. The cell extracts were centrifuged at 5,000 × *g* for 10 min at 4°C to remove the organic phase, followed by centrifugation at 20,000 × *g* for 10 min at 4°C to remove cell debris. The resulting supernatants were analyzed immediately using HPLC-MS as described previously (12, 25).

For detection of metabolites during growth in minimal media, fresh colonies of X. campestris pv. *campestris* wild type, Δ*sah* mutant, and Δ*sah*::P*sah* complementation strain were inoculated into 25 mL MOPS EZ defined medium (Teknova) without 10× ACGU and with 0.2% glucose replaced by 0.2% glutamate. Cultures were incubated at 28°C with shaking (200 rpm) to reach an OD_600_ of ≈0.2. Twenty-milliliter cultures were harvested for metabolite extraction and detection using HPLC-MS (12, 25).

Metabolomic data analysis was performed using the Metabolomics Analysis and Visualization Engine (MAVEN) software (60). Ion counts of metabolites were normalized by the OD_600_ and unit volume of the culture. Estimation of cellular NADP(H) and NAD(H) concentrations was done as described previously (25) with the detection efficiency of NADPH in LC-MS set as 3.02e7 ion counts/μM in a 25-μL sample, as well as an estimated X. campestris pv. *campestris* cell volume of 2.377 fL.

### Bacterial growth measurement.

For growth measurement, fresh colonies of X. campestris pv. *campestris* wild type, Δ*sah*, and Δ*sah*::P*sah* strains on LB agar were resuspended into growth media and diluted to an OD_600_ of ≈0.005 in 96-well plates. Growth was monitored by the OD_600_ at 28°C under shaking in a Synergy2 microplate reader (BioTek). Doubling times were estimated by fitting the growth data to the exponential growth curve using a custom python script. For counting CFU, X. campestris pv. *campestris* cultures were serially diluted and plated on LB agar. Colonies were counted after incubation at 28°C for 2 to 4 days.

### Plant pathogenicity assay.

Pathogenicity of X. campestris pv. *campestris* wild type and Δ*sah* mutant was assessed by infection on radish (Raphanus sativus L. cv. Japan 501) leaves. Twenty-microliter aliquots of cell suspensions (OD_600_, 0.3) were injected into the main vein on the backs of leaves of radish seedlings at the 5-true-leaf stage. A negative control was prepared by injecting an equivalent volume of sterile 0.85% NaCl. Symptoms of disease were evaluated at 7 days postinoculation (dpi). For reisolation of bacteria from infected leaves, 3 leaf disks (9 mm) were collected from each inoculated leaf using a hole puncher. The leaf disks were disinfected by immersion in 1% sodium hypochlorite (NaClO) solution for 1 min, washed twice using sterilized water, and snap-frozen using liquid nitrogen. The frozen samples were then homogenized using a Retsch MM400 ball-milling machine at 60 Hz for 1 min. The resulting leaf powder was resuspended in 0.85% NaCl followed by serial dilution and plating on LB agar. The plates were incubated at 28°C for 2 days followed by colony counting.

### Biofilm and exopolysaccharide assay.

Biofilm production by X. campestris pv. *campestris* was measured using the crystal violet method adapted for *Xanthomonas* (61). Fresh X. campestris pv. *campestris* colonies were inoculated into LB at an OD_600_ of 1 and incubated at 28°C without shaking to promote biofilm growth. After a 5-day incubation, nonadherent cells and culture media were removed by decanting. The adherent biofilm was stained with 0.1% (wt/vol) crystal violet solution for 30 min, then washed twice with sterilized water. The biofilm-bound crystal violet was solubilized in absolute ethanol and quantified by absorbance at 600 nm.

EPS production by X. campestris pv. *campestris* strains was measured using LB agar containing 2% glucose, as described previously (62). Two-microliter aliquots of X. campestris pv. *campestris* cell suspensions (OD_600_, 0.3) were patched onto LB-glucose plates and incubated at 28°C for 4 days. EPS production was observed by comparing the mucoidiness of the patches and the diameters of colonies.

### X. campestris pv. *campestris* soil survival assay.

Survival of X. campestris pv. *campestris* in the field was conducted in soil sampled from a vegetable patch planted with Chinese cabbage in 5 consecutive years. Overnight culture of wild type and Δ*sah* (OD_600_, 0.3) was inoculated into 5 g unsterilized soil for each time point (0, 1, 2, 3, and 5 days). To recover X. campestris pv. *campestris* from soil, the X. campestris pv. *campestris*-inoculated soil was added into a 250-mL flask with 30 mL 0.85% NaCl under shaking for 30 min and then the suspension was filtered by 4-layer cheese cloth. The filtered suspension was serially diluted and plated on selective mCS20ABN media (63) containing 2 g/liter soya peptone, 2 g/liter Bacto tryptone, 1.59 g/liter KH_2_PO_4_, 0.33 g/liter (NH_4_)_2_HPO_4_, 0.4 g/liter MgSO_4_·7H_2_O, 6 g/liter l-glutamine, 1 mL/liter 0.75% methyl green (adjusted to pH 6.5 with NaOH), 12.5 g/liter soluble starch, and 15 g/liter Bacto agar) with 50 μg/mL rifampin. The plates were incubated at 28°C for 2 days followed by colony counting to enumerate the X. campestris pv. *campestris* titer in the soil.

### Interspecies competition assay.

To measure survival of X. campestris pv. *campestris* against Pseudomonas, 1 mL of Pseudomonas aeruginosa strain PA01 or Pseudomonas sp. strain ADAK18 (45) in LB (OD_600_, 2.0) was mixed with 100 μL of X. campestris pv. *campestris* (WT, Δ*sah*, or Δ*sah*::P*sah*) (OD_600_, 1.0). Aliquots of 10 μL of each competition mixture were patched on 3% LB agar plates in triplicate and incubated face up at 28°C for 24 h. At 0 h and 24h, the competition spots were excised from the agar plate and fully resuspended in LB, followed by serial dilution and plating on X. campestris pv. *campestris*-selective plates (LB agar containing 50 μg/mL rifampin and 5 μg/mL tetracycline). The plates were incubated at 28°C for 2 days followed by colony counting.
