# Supplementary figures and images for: Metabolic Promiscuity of an Orphan Small Alarmone Hydrolase Facilitates Bacterial Environmental Adaptation
Source: mBio. 2022 Dec 6;13(6):e02422-22. doi: 10.1128/mbio.02422-22 (PMC9765508; doi:10.1128/mbio.02422-22)

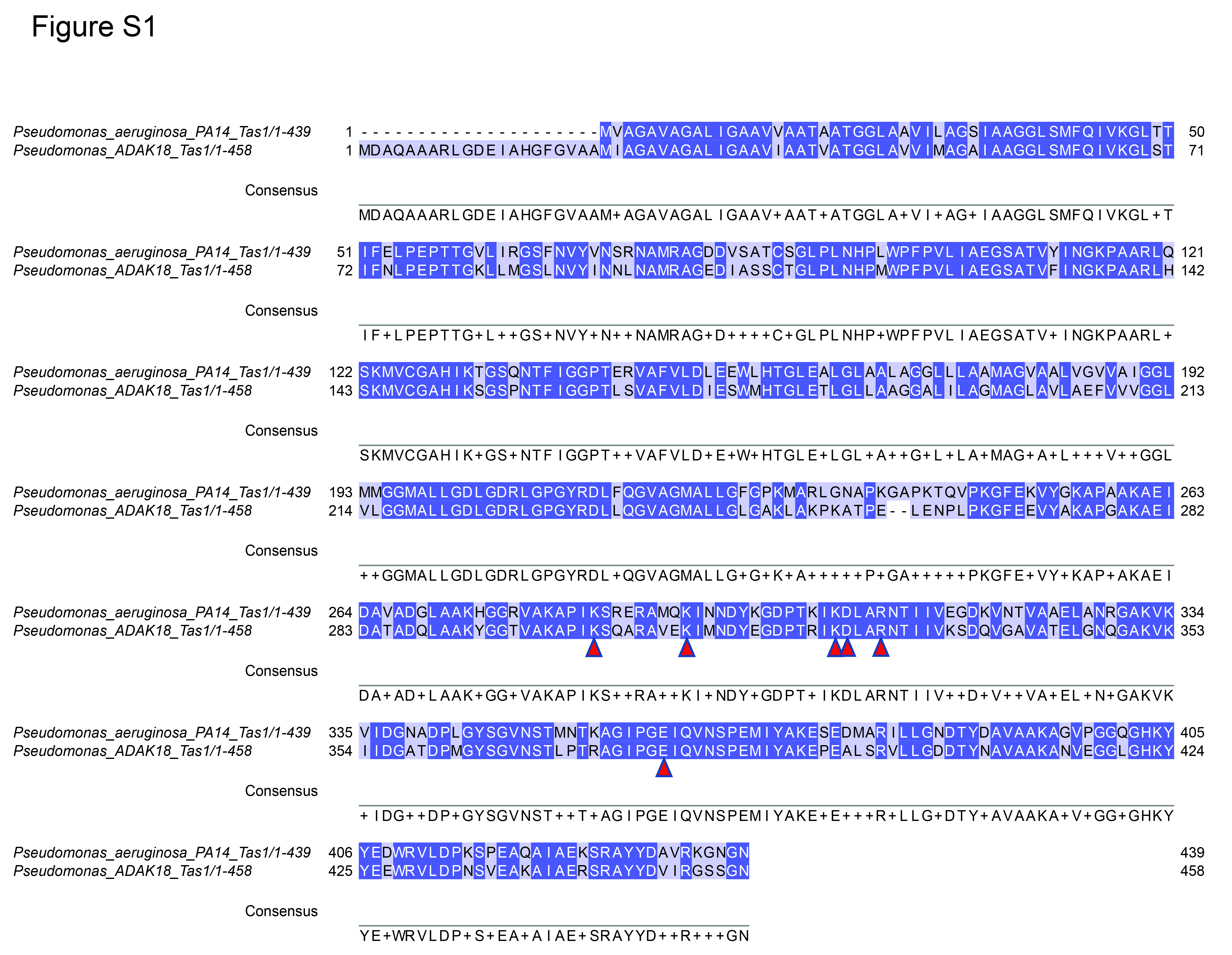

Supplement: FIG S1 [file mbio.02422-22-s0001.tif]

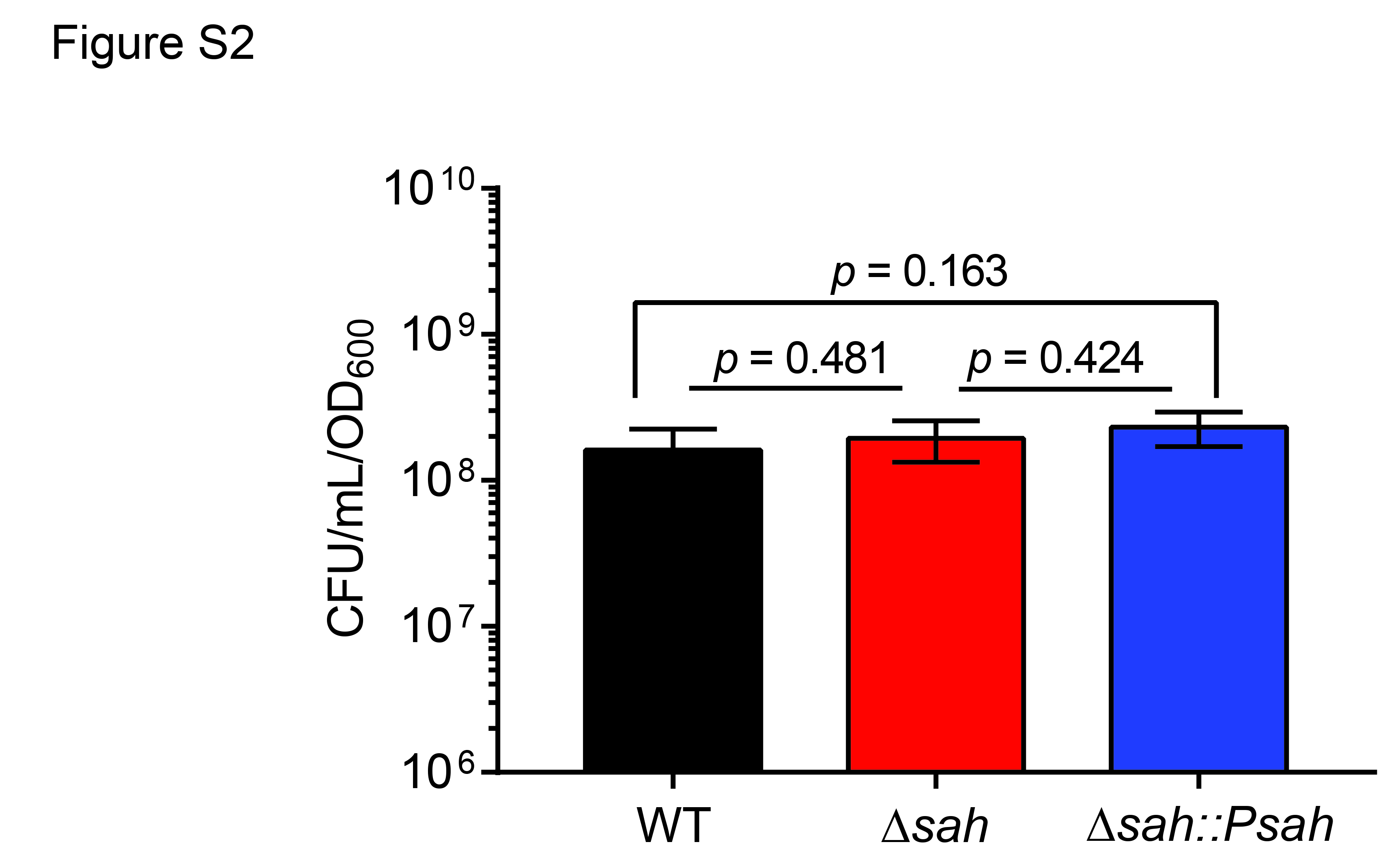

Supplement: FIG S2 [file mbio.02422-22-s0002.tif]

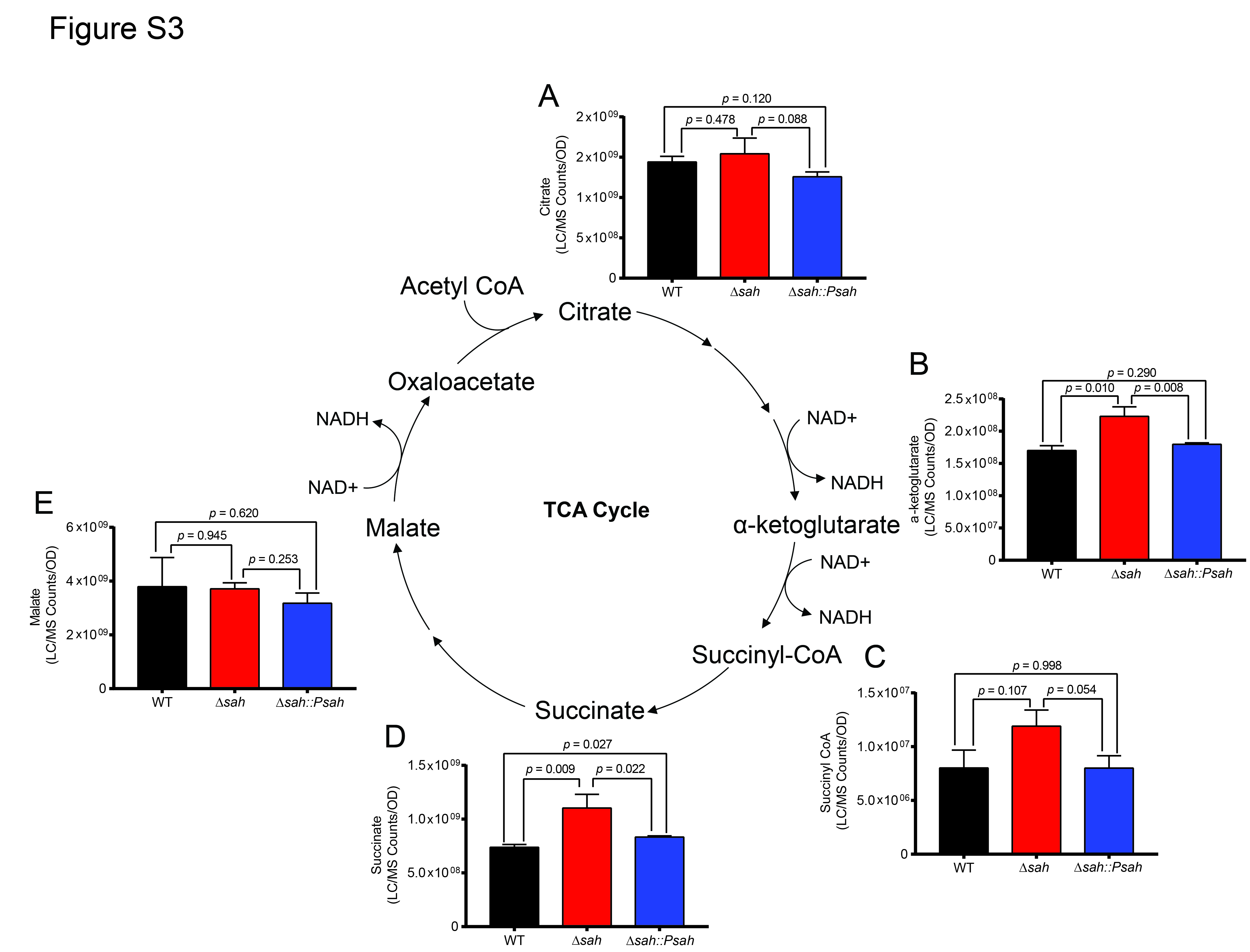

Supplement: FIG S3 [file mbio.02422-22-s0003.tif]
